# Epididymitis

**Published:** 1912-12

**Authors:** 

**Affiliations:** Atlanta, Ga.; Atlanta, Ga.


### EPIDIDYMITIS

By Ballenger & Elder, Arlanta, Ga.

Epiddiymitis is an inflammation of the epididymis and is a frequent complication of gonorrheoa. It occurs most frequently between the second and fourth week of the disease, but may be brought on earlier by indiscretions of various kinds, or may come at any time during chronic infection with gonococci or other organisms.

The exciting cause may be any one or more of the following: Strong irritating injections, the passage of urethral instruments, alcoholic or venereal excesses, sexual excitement. physical exertion and taumatism of the epididymis or genital structures. The gonococci are carried back or spread to the deeper parts and pass through the seminal ducts, the ampullations of the vas deferens, and through the cord to the epididymis. Inflammation of the deep urethra and seminal vesicles may or may not cause sufficient trouble to attract attention. It is claimed that a reverse peristalsis may take the germs along rapidly, and without their attacking the seminal duct, vesicle or cord. Judicious treatment with irrigations of the urethra and bladder tend to prevent rather than to cause the development of epididymitis.

Premonition of epidily is sometimes manifested by pain in the seminal vesicles or in the deep urethra. Occasionally pain or soreness is felt in the lower abdomin just above the groin. This may gradually extend down the vas to the epididymis. More frequently no premonitory symptoms are noticed. The epididymis begins to swell and ache, and gradually becomes very tender; the discharge as a rule ceases, but it returns later; there may be a chill and fever, malais, loss of appetite and occasionally severe symtemic reaction. The swelling is at first limited to the epididymis, although subsequently, the testicle or the cord between the epididymis and the ingunial ring may become involved. There may be a collection of fluid in the tunica vaginalis. The mass may reach the size of an ordinary fist, the skin of the scrotum may be red and edematous. The pain on movement or pressure gradually subsides, the swelling becomes softer and finally disappears, except a nodule in the epididymis, which may persist for some time. In rare cases a large abscess may form, and, still more rarely gangrene of the scrotum may occur. Chronic infection may persist causing recurrent attacks of epididymitis: such infection is, as a rule, non-gonorrhoeal. Neuraligia of the testicle may follow, or there may be a sensitiveness of this organ and the epididymis in neuropathic subjects.

The diagnosis rarely involves any uncertainty, if a careful examination is made and the acute course of the disease, together with the above symptoms, is considered. Tuberculosis may originate in the epididymis and cause the production of a chronic epididymitis, less acute and less painful than gonorrhoeal.

The prognosis is good, as resolution usually occurs promptly, and more or less completely, in from one to four weeks, under appropriate treatment. Sterility is very likely to follow double epdidymitis. The danger of recurrences depends upon the success in the treatment of the deep seated urethral, prostatic, or vesicular inflammation.

Treatment of epididymitis requires, generally speaking, rest in bed, cold or heat and suspension of the inflamed organ. The swollen part should be kept well up on the symphysis pubis by a suspensory or upon a shelf of adhesive plaster placed across the thighs, the upper border of the plaster reaching to the perineum.

All local urethral treatment should be discontinued during the acute part of the epididymitis has subsided. The application of an ointment composed of guaiacol 15% and ichthyol 30% in lanoline and vaseline aid in the reduction of the inflammation and in the relief of the pain. The ice bag also greatly reduces both the swelling and the pain. It should be only partly filled with shaved or finely crushed ice, and the air expelled from it just before the cap is screwed tightly on. The ice bag should be carefully adjusted over the epididymis which is kept well up on the symphysis pubis and should not be allowed to drag down between the legs. The guaiacol and ichthyol ointment should be used in conjunction with the ice bag and may be applied twice daily and covered with oil silk to protect the clothing and ice bag from the ointment.

If preferred hot poultices or the hot water bag may be used instead of the ice, but in our experience the cold application is more comfortable and more prompt in the reduction of the swelling. Vaccine therapy may be resorted to and occasionally will be followed by satisfactory results at other times negative results. The dose need not be less than 50 million gonococci every two or three days. The patient is usually able to get out of the bed in from one to two weeks. occasionally earlier. During the subsiding stage iodide of potash in doses of ½ gram (7½ grains) to 1 gram (15 grains) three times daily, is administered to hasten the absorption of the infiltration of the epididymis. Snug support in a suspensory or strapping the swollen part with adhesive plaster so as to compress it uniformly will cause a more rapid return to normal and lessen the danger of a relapse.

Hagner's Epididymotomy.—A rational treatment of epididymitis has been suggested by Hagner which consists of incising the swollen epididymitis and draining the small abscesses. This operative treatment is not indicated in all cases but only in those where the pain is severe and does not respond promptly to rest and local applications or where there are recurrent attacks. It is further indicated where the patient is particularly anxious to be up and around at the earliest possible time.

Technic.—The scrotum, pubic and perineal regions should be shaved and scrubbed the night before the operation, and a moist 1 to 5,000 bichloride of mercury dressing applied. A cathartic should be given the evening before and an enema a few hours before the operation. The scrotum should again be washed with bichloride and alcohol immediately preceding the operation. If preferred a 2% solution of iodine in alcohol may be applied after shaving and washing with alcohol. A loose dry sterile dressing should be used after the iodine has thoroughly dried instead of the moist bichloride dressing as the iodine is not effective on the moist skin. Another application should be made just before operating.

As only a few minutes are required for the operation, nitrous oxide gas and oxigen anesthesia is sufficient. The testicle should be turned upward in such a manner that the incision may be made in the lower posterior part, so as to avoid the tunics vaginalis, and while being firmly grasped the scalpel is carried through the skin and facias directly into the epididymis. This can be done without changing the hold on the testicle and scrotum. The distinct modules in the globus major, minor or wherever they can be detected are opened. Only a small amount of pus, as a rule, is found. The skin incision need be only about ¾ of an inch in length and requires no sutures. A drain of iodoform gauze is placed in the incision and should be removed in 24 to 36 hours. If hydrocele is present it should be tapped. Sterile gauze and cotton should be applied and held in place by a Peterkin scrotal bandage.

The patient should remain in bed five to seven days. The dressings should be changed every other day. A snug fitting suspensory should be worn when the patient gets up. The incision is allowed to drain without interference until it heals; this will occur in from one to two weeks. The pain is relieved at once by the operation but the swelling and induration require ten days or two weeks in which to undergo resolution.

A saturated solution of nitrate of silver in pure carbolic acid applied just within the incision cauterizes it and prevents its healing too promptly.

Ashcraft has modified the operation of Hagner and makes the incision through the lower anterior portion of the scrotum into the tunica vaginalis and in this manner exposes the epididymis with its many small pockets of pus. These are well opened and the incision in the scrotum is partly closed with cat-gut sutures. Iodoform gauze is placed in the wound for drainage. The after treatment is practically the same for both operations, though Ashcraft recommends that the gauze drainage be replaced several times.

